# Binocular treatment of amblyopia using videogames (BRAVO): study protocol for a randomised controlled trial

**DOI:** 10.1186/s13063-016-1635-3

**Published:** 2016-10-18

**Authors:** Cindy X. Guo, Raiju J. Babu, Joanna M. Black, William R. Bobier, Carly S. Y. Lam, Shuan Dai, Tina Y. Gao, Robert F. Hess, Michelle Jenkins, Yannan Jiang, Lionel Kowal, Varsha Parag, Jayshree South, Sandra Elfride Staffieri, Natalie Walker, Angela Wadham, Benjamin Thompson, on behalf of the BRAVO study team, Taina Von Blaramberg, Stephen J. Boswell, Arijit Chakraborty, Lily Chan, Simon Clavagnier, Patrick W. C. Ho, Colin Howe, Michelle H. Jen, Lisa Kearns, Joanna Michie, Colleen Ng, John Faafetai Faatui, Peter Pang, Roberto Pieri, Rajkumar Nallour Raveendren, Daniel Spiegel, Stuart L. Uren

**Affiliations:** 1 School of Optometry and Vision Science, The University of Auckland, Auckland, New Zealand; 2 School of Optometry and Vision Science, University of Waterloo, Waterloo, ON Canada; 3 School of Optometry, Faculty of Health and Social Sciences, The Hong Kong Polytechnic University, Hong Kong, SAR China; 4 Department of Ophthalmology, Starship Children’s Hospital, Auckland, New Zealand; 5 Department of Ophthalmology, McGill Vision Research, McGill University, Montreal, QC Canada; 6 National Institute for Health Innovation, School of Population Health, The University of Auckland, Auckland, New Zealand; 7 Department of Surgery, Centre for Eye Research Australia, Royal Victorian Eye and Ear Hospital; Ophthalmology, University of Melbourne, Melbourne, VIC Australia

**Keywords:** Amblyopia, Binocular vision, Suppression, Plasticity, Videogame, Perceptual learning

## Abstract

**Background:**

Amblyopia is a common neurodevelopmental disorder of vision that is characterised by visual impairment in one eye and compromised binocular visual function. Existing evidence-based treatments for children include patching the nonamblyopic eye to encourage use of the amblyopic eye. Currently there are no widely accepted treatments available for adults with amblyopia. The aim of this trial is to assess the efficacy of a new binocular, videogame-based treatment for amblyopia in older children and adults. We hypothesise that binocular treatment will significantly improve amblyopic eye visual acuity relative to placebo treatment.

**Methods/design:**

The BRAVO study is a double-blind, randomised, placebo-controlled multicentre trial to assess the effectiveness of a novel videogame-based binocular treatment for amblyopia. One hundred and eight participants aged 7 years or older with anisometropic and/or strabismic amblyopia (defined as ≥0.2 LogMAR interocular visual acuity difference, ≥0.3 LogMAR amblyopic eye visual acuity and no ocular disease) will be recruited via ophthalmologists, optometrists, clinical record searches and public advertisements at five sites in New Zealand, Canada, Hong Kong and Australia. Eligible participants will be randomised by computer in a 1:1 ratio, with stratification by age group: 7–12, 13–17 and 18 years and older. Participants will be randomised to receive 6 weeks of active or placebo home-based binocular treatment. Treatment will be in the form of a modified interactive falling-blocks game, implemented on a 5th generation iPod touch device viewed through red/green anaglyphic glasses. Participants and those assessing outcomes will be blinded to group assignment. The primary outcome is the change in best-corrected distance visual acuity in the amblyopic eye from baseline to 6 weeks post randomisation. Secondary outcomes include distance and near visual acuity, stereopsis, interocular suppression, angle of strabismus (where applicable) measured at baseline, 3, 6, 12 and 24 weeks post randomisation. Treatment compliance and acceptability will also be assessed along with quality of life for adult participants.

**Discussion:**

The BRAVO study is the first randomised controlled trial of a home-based videogame treatment for older children and adults with amblyopia. The results will indicate whether a binocular approach to amblyopia treatment conducted at home is effective for patients aged 7 years or older.

**Trial registration:**

This trial was registered in Australia and New Zealand Clinical Trials Registry (ACTRN12613001004752) on 10 September 2013.

## Background

Amblyopia is a neurodevelopmental disorder of the visual system that is caused by abnormal binocular visual experience during early childhood, typically due to anisometropia or strabismus [1]. Amblyopia causes a range of monocular deficits in the affected eye including impaired visual acuity [1], contrast sensitivity [2], motion perception [3, 4] and excessive crowding [5] (see [6] for a recent review). Patients with amblyopia also experience impaired binocular vision. In particular, the fellow eye often suppresses the amblyopic eye when both eyes are open, and stereopsis is commonly impaired or absent [7]. Stronger interocular suppression has been associated with poorer stereopsis and monocular visual acuity in patients [8–11], as well as poorer amblyopic eye contrast sensitivity in animal models of amblyopia [12, 13].

In children, the visual acuity deficit associated with amblyopia can be treated monocularly by optically correcting any significant refractive error and then occluding (patching) or penalising (with atropine cycloplegia) the fellow eye to encourage use of the amblyopic eye [14]. While effective, these monocular treatments may result in adverse psychosocial effects [15–17] and compliance can be low [18]. In addition, patching and atropine have relatively high relapse rates after cessation of treatment [19, 20], and they do not directly address binocular deficits that are associated with amblyopia [21]. Therefore, residual monocular and binocular visual impairments often remain after patching and/or atropine therapy [22].

Treatment of teenagers and adults with amblyopia is also problematic. Conventional treatment can be effective in teenagers [22] and a large number of laboratory-based studies have reported that visual function can be improved in adults with amblyopia using techniques such as monocular perceptual learning (reviewed by [23]). However, despite this evidence, teenagers and adults with amblyopia are typically left untreated in mainstream clinical practice. This may be because older patients are assumed to be unable to improve, are not able to tolerate conventional treatments, or because clinical trials have not assessed the treatment of amblyopia in adults. There is a clear need for clinicians to be provided with alternative treatment options for patients of all ages that are supported by high-quality clinical trial data [24–26].

In recent years a new binocular amblyopia treatment approach has emerged [27] that is designed to reduce suppression and strengthen binocular visual function [26, 28]. Binocular treatment is based on evidence that patients with amblyopia have the ability to combine information between their eyes if suppression is minimised by presenting stimuli at high contrast to the amblyopic eye and at low contrast to the fellow eye (contrast balancing) [29, 30]. This indicates that patients with amblyopia may have a structurally intact, but functionally suppressed, binocular visual system. Binocular treatment involves tasks that require binocular combination of stimuli that are presented dichoptically with a contrast offset in favour of the amblyopic eye. As treatment progresses, the interocular contrast difference is gradually reduced to promote binocular fusion.

The first contrast balanced binocular treatment studies involved repeated performance of a psychophysical motion discrimination task under dichoptic presentation conditions [27, 31]. Subsequent studies employed a falling-blocks videogame that requires blocks moving down the screen to be tessellated together [32]. The videogame can be played using a pair of video goggles that allow for separate images to be presented to each eye [33, 34] or on a tablet computer device with images split between the two eyes using a lenticular overlay screen or red/green anaglyphic glasses [32, 35, 36]. Neither eye sees all of the game elements and, therefore, binocular combination is required for successful game play.

There have been several case-series studies investigating the effect of contrast balanced binocular treatment in adults and children with amblyopia and the results are promising [27, 31, 32, 34, 35, 37–42]. A recent review of these studies reported an average improvement in amblyopic eye visual acuity of 0.24 logarithm of the minimum angle of resolution (LogMAR) for adults (*n* = 84) and 0.16 LogMAR in compliant children (*n* = 91); significant improvements in stereopsis were also observed in both adults and compliant children [26]. More recently, binocular treatment has been reported to improve fine motor skills in a case-series of children with amblyopia [43]. Furthermore, viewing of dichoptic, contrast balanced movies was found to improve amblyopic eye visual acuity by 0.2 LogMAR in a group of children [44]. As a whole, the results from preliminary studies suggest that (1) contrast balanced binocular treatment improves monocular and binocular visual function in children and adults, (2) the treatment is rapid, working within a matter of weeks and (3) the effects last in excess of 1 month after the cessation of treatment. The momentum behind these initial studies has led to multiple independent calls for a formal, randomised clinical trial of the treatment [45, 46], which we hope to answer with this study.

A number of other binocular amblyopia treatment approaches are in development (e.g. [47, 48]). Some are also based on the use of contrast balancing, such as a modified dichoptic first-person shooter videogame combined with a monocular perceptual learning task [49]. Others, such as the I-BiT system [50, 51], involve dichoptic presentation of images without contrast balancing. The I-BiT system involves the dichoptic presentation of videos or videogames with the background presented to both eyes and foreground elements presented only to the amblyopic eye. The treatment is targeted at children and aims to improve amblyopic eye visual acuity.

A 2015 Cochrane Review reported no published randomised controlled trials of any binocular intervention in children with unilateral amblyopia aged 3–8 years [52]. Subsequently, a randomised controlled trial (RCT) evaluating of the effectiveness of the I-BiT system was completed [53]. The trial compared the effect of 3 h (30 min per week) of dichoptic videos versus dichoptic videogames versus nondichoptic (control) videogames on amblyopic eye visual acuity in children aged 4–8 years. Amblyopic eye visual acuity improved by approximately 0.07 LogMAR in all three arms [53].

In addition, a RCT of contrast balanced binocular treatment was recently completed [54]. This trial used an adventure videogame called Dig Rush rather than the falling-blocks game described above. Children of 4–10 years of age completed 10 h of binocular treatment (1 h per day, 5 days per week) and 28 h of patching (2 h per day, 7 days per week) following a randomised crossover design. Contrast balanced binocular treatment resulted in a significantly greater amblyopic eye visual acuity improvement than patching (0.15 LogMAR versus 0.07 LogMAR prior to crossover). A large-scale RCT comparing contrast balanced binocular treatment delivered using the falling-blocks game to patching (NCT02200211) will also be complete in the near future.

Aside from the studies described above, a literature search completed on 25 September 2016 using the same search strategy employed within the 2015 Cochrane Review by Tailor et al. [52] revealed no additional, published RCTs using a binocular intervention for either children or adults with unilateral amblyopia.

## Methods/design

### Aim and hypothesis

The primary aim of this trial is to investigate whether 6 weeks of binocular treatment leads to a greater improvement in amblyopic eye visual acuity than 6 weeks of a placebo treatment. The trial will also assess whether binocular treatment improves stereopsis and quality of life, and reduces interocular suppression to a greater extent than placebo treatment. Furthermore, compliance and participant acceptability of the home-based videogame treatment will also be addressed. We hypothesise that binocular treatment will lead to greater improvements in monocular (near and distance visual acuity) and binocular (stereopsis and suppression) visual function after 6 weeks of treatment compared to placebo treatment.

### Study design and recruitment

This is a double-blind, randomised, placebo-controlled multicentre trial. Five trial sites are involved, namely: School of Optometry and Vision Science, the University of Auckland, Auckland, New Zealand; School of Optometry and Vision Science, University of Waterloo, Waterloo, Canada; The Centre for Eye Research Australia, Royal Victorian Eye and Ear Hospital, Melbourne, Australia; McGill Vision Research, Department of Ophthalmology, McGill University, Montreal, Canada; and the School of Optometry, Hong Kong Polytechnic University, Hong Kong. Data will be collected in both clinical and psychophysical laboratory settings depending on the facilities available at each site.

Potential participants will be identified through referrals by ophthalmologists and optometrists, clinical record searches and public advertisements. Potential participants (and parents/guardians of potential child participants) will contact a study team member and will be provided with an information sheet. Interested participants will complete a telephone screening and, if appropriate, will be invited for an eligibility and baseline assessment by the study staff. Recruitment began on 13 March 2014 and is scheduled to end on the 31 May 2016.

### Eligibility

The following eligibility criteria exist for this trial:

- Age 7 years or older
- Amblyopia associated with the presence or history of strabismus, anisometropia or both (mixed mechanism)
- Unilateral amblyopia, defined as best-corrected amblyopic eye visual acuity (VA) of 0.30–1.00 LogMAR inclusive, fellow eye VA ≤0.10 LogMAR and an interocular VA difference ≥ 0.20 log units. VA will be measured using the electronic Early Treatment for Diabetic Retinopathy Study (E-ETDRS) protocol presented on an Electronic Visual Acuity (EVA) testing system [55]
- Strabismic amblyopia, defined as amblyopia in the presence of heterotropia at distance and/or near fixation, a history of strabismus surgery, or resolution of strabismus following hyperopic spectacle correction
- Anisometropic amblyopia, defined as amblyopia in the presence of a spherical equivalent difference ≥0.50 dioptres (D) between the eyes, or a difference of astigmatism in any meridian ≥1.50 D and no strabismus
- Mixed-mechanism amblyopia, defined as amblyopia in the presence of both strabismus and anisometropia
- An ability to align a dichoptically presented nonius cross within the screen area of an iPod touch device. This criterion ensures that successful play of the treatment game is possible
- Willing and able to provide written informed consent for participation in the study
- Stable visual acuity with full optical correction

Exclusion criteria are: myopia of spherical equivalent power more than −6.00 D in either eye; previous intraocular surgery; ocular pathology; a diagnosed neurological condition (judged on a case-by-case basis by the Trial Steering Committee).

#### Optical treatment

Participants will be required to wear their full optical correction in spectacles or contact lenses prior to randomisation. Optical correction will meet the criteria designed by the Paediatric Eye Disease Investigator Group and will be based on a cycloplegic refraction that is within 6 months of study entry (a new cycloplegic refraction will be provided if necessary): (1) hypermetropia will not be under-corrected by more than +1.50 D spherical equivalent and the reduction in plus sphere must be identical between the two eyes, (2) spherical equivalent power will be ≤ ±0.50 D of fully correcting the anisometropia, (3) cylinder power in each eye will be ≤ ±0.50 D of fully correcting the astigmatism for each eye and, (4) cylinder axis of the correction for each eye must be ≤ ±6° of the axis identified during cycloplegic refraction when cylinder power is ≥1.00 D.

Participants who have worn full optical correction full-time for more than 16 weeks and meet all eligibility criteria will be randomised immediately after baseline examination. Participants who have not been wearing full optical correction full-time for 16 weeks but are otherwise eligible will complete an optical treatment period of up to 16 weeks before randomisation. If they remain eligible, participants undergoing optical treatment will be randomised when the visual acuity in the amblyopic eye is stable, defined as a change of ≤0.1 LogMAR over two measurements made at least 4 weeks apart. Participants will not be eligible if their visual acuity does not stabilise over 16 weeks of refractive adaptation, or if they are unable to wear optical correction full-time, or if their amblyopic eye visual acuity improves to the point that they no longer meet the eligibility criteria. Randomised participants will be asked to wear their optical correction full-time until their final follow-up visit is complete. This aspect of the protocol is designed to minimise the effect of optical treatment on the outcome measures of the trial.

Written informed consent will be obtained and eligibility assessed at the baseline visit. Participants who are eligible will be randomised and begin treatment. Participants who require optical treatment but are otherwise eligible will be provided with optical correction and enter the optical treatment process. Participants who remain eligible after optical treatment will be randomised and the measurements made at the end of optical treatment will be used as the pretreatment baseline.

#### Randomisation and blinding

Allocation concealment procedures will be followed. Eligible participants will be allocated randomly in a 1:1 ratio by computer to either the active or placebo treatment group, using minimisation stratified by three age groups (7–12, 13–17 and 18 years and over). Participants and study investigators will remain blinded to treatment allocation during the study, as will all clinical examiners/testers involved in recruitment, data collection and data entry until final data lock. Independent study staff responsible for randomisation and allocation of the game-specific device to participants will not be blinded. Participants will be informed of the study results at the end of the study and participants randomised to the placebo group will be offered the treatment if the trial is positive.

There is a possibility that participants may attempt to guess their allocated group (active or placebo) by observing the game and, therefore, become unblinded. A number of steps will be taken to minimise this risk. No details regarding the principles underlying the game design, such as contrast balancing or the presentation of different game elements to each eye, will be provided to participants. Also, study staff will be instructed not to answer questions from participants relating to their allocated group. In addition, study clinicians who assess treatment outcomes will be instructed not to handle the training iPod to prevent accidental unblinding.

#### Treatment allocation

**Fig. 1:**
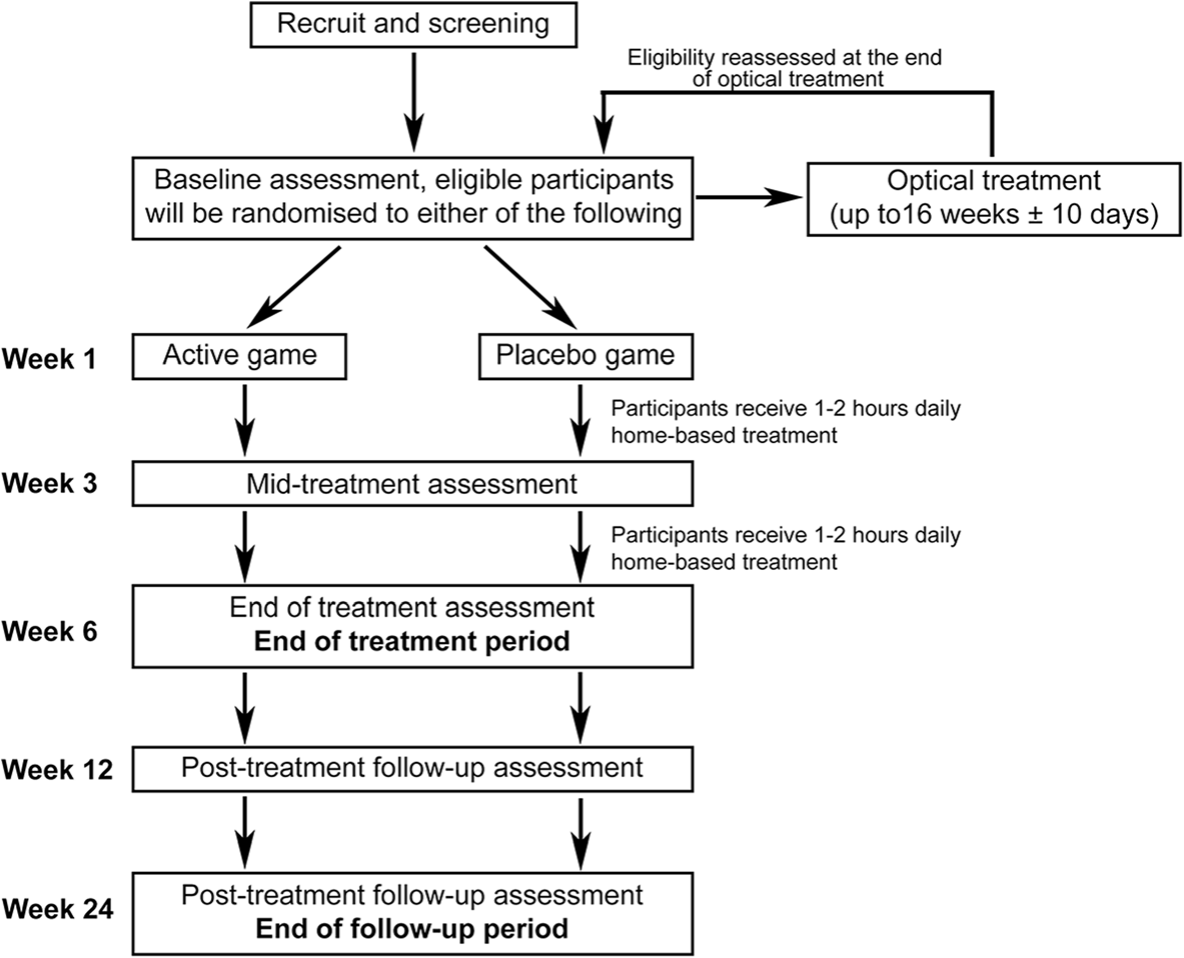
Study schematic. Legend: study flow from recruitment and screening to the final 24-week follow-up

Participants will be randomised to receive 6 weeks of active or placebo home-based binocular treatment in the form of an interactive falling-blocks game implemented on a 5th generation iPod touch device viewed through red/green anaglyphic glasses (Fig. 1). Participants will begin their treatment within 1 day of randomisation. Participants will be instructed to play the videogame for 1–2 h every day (separated into a maximum of three daily sessions) for 6 weeks while wearing red/green anaglyphic glasses and their optical correction where applicable. The treatment will terminate automatically on the iPod 44 days after randomisation, regardless of the scheduled follow-up clinical visit, to ensure consistent dosing for all participants. This trial uses a 6-week treatment period as previous studies indicate that this duration is sufficient for significant improvements [35, 37, 39, 40] and that compliance is high early in therapy but begins to fall after 6 weeks if the game is not changed.

##### Active group

In the active treatment game, a subset of blocks is presented to the amblyopic eye at high contrast (100 %) and other blocks are presented to the fellow eye at low contrast tailored to each individual’s level of interocular suppression determined by a random-dot kinematogram measure of suppression [30, 33] and an assessment of the participant’s ability to play the game by an unblinded assessor. Red-green anaglyphic glasses (in addition to spectacle correction, if any; green lens over the amblyopic eye) are used to split the images between the two eyes. The contrast of the blocks presented to the fellow eye will be increased if participants play the game successfully. Specifically, if the patient achieves a score of 1000 and played for more than 15 min within a 24-h period, the fellow eye contrast will be increased by 15 % for the next 24-h period of play. If the game is attempted for over 15 min but a score of 1000 is not achieved, the fellow eye contrast will be reduced by 5 %. In all other situations the fellow eye contrast will remain constant.

##### Control group

The placebo treatment game will be identical to the active treatment game except that both eyes will see the same images with no contrast offset. All participants will be required to wear red-green anaglyphic glasses on top of their optical correction during game play.

Both the treatment and placebo games include a dichoptically presented, contrast-balanced alignment calibration at the start of each treatment session. The participants will be presented with a dichoptic nonius cross that they are required to align using directional arrow buttons presented on the iPod screen. In the active game, the alignment information will be used to offset the relative position of the blocks shown to each eye to ensure proper alignment during game play.

The iPod devices will automatically record treatment information including contrast change, alignment calibration, scores achieved and compliance (time spent playing the game).

#### Outcomes

Outcome measures will be assessed at 3, 6, 12 and 24 weeks post randomisation by blinded assessors. Baseline and outcome measures are summarised in Table 1.

**Table 1:** Schedule of assessments

	Randomisation	3 weeks	6 weeks	12 weeks	24 weeks
Demography	√				
Visual acuity	√	√	√	√	√
Stereopsis	√	√	√	√	√
Angle of strabismus (if applicable)	√	√	√	√	√
Interocular suppression	√	√	√	√	√
Interocular contrast		√	√		
Treatment compliance		√	√		
Treatment acceptability		√	√		
Quality of life (18 years and over)	√				√

#### Primary outcome

The primary outcome is the change in best-corrected distance visual acuity in the amblyopic eye from baseline to 6 weeks post randomisation, measured using the highly standardised E-ETDRS protocol EVA testing system [55].

#### Secondary outcomes

- Change from baseline in best-corrected distance visual acuity in the amblyopic eye, fellow eye and both eyes at 3, 6, 12 and 24 weeks post randomisation
- Change from baseline in best-corrected near visual acuity in the amblyopic eye, fellow eye and both eyes at 3, 6, 12 and 24 weeks post randomisation measured using the Lighthouse ETDRS near visual acuity chart
- Change from baseline in stereopsis at 3, 6, 12 and 24 weeks post randomisation measured using Randot Preschool Stereotest [56]
- Change from baseline in angle of strabismus (where applicable) at 3, 6, 12 and 24 weeks post randomisation measured using the Simultaneous prism Cover Test at near and distance [22]
- Change from baseline in interocular suppression at 3, 6, 12 and 24 weeks post randomisation measured using Worth Four-dot Test at 33 cm and at 6 m and a prototype iPod-based version of the Dichoptic Motion Coherence Test [8, 10, 33]
- Change from baseline in interocular contrast at 3 and 6 weeks post randomisation based on the log file extracted from the iPod
- Change in quality of life from baseline to 24 weeks post randomisation measured in adult participants only (18 years of age or older) using the World Health Organisation Quality of Life (WHOQOL) – BREF [57]
- Treatment compliance at 3 and 6 weeks post randomisation. Participants’ compliance will be recorded in two ways: through data stored on the iPod device and through a diary provided to participants. Compliance will be defined as at least 25 % of the prescribed dose (i.e. playing the game for at least 5 h 15 min at 3 weeks and for at least 10 h 30 min at 6 weeks post randomisation) [37]
- Treatment acceptability at 3 and 6 weeks post randomisation, assessed using a modified version of the Amblyopia Treatment Index questionnaire for children and adults [58, 59]. This questionnaire has 5-point Likert Scales for a series of 20 questions for parents of participants aged 7–17 years and 19 questions for participants aged 18 years and over
- Adverse events. No cases of diplopia have been reported in published studies of contrast balanced binocular treatment, possibly because the treatment is designed to promote binocular function. However, diplopia is an important issue for any amblyopia treatment. Two cases of diplopia related to treatment using the I-BiT system have been reported and both cases resolved spontaneously following cessation of treatment [53]. Therefore, binocular treatment may carry a risk of diplopia. The potential side effect of diplopia will be monitored throughout the study. Diplopia will be discussed upon randomisation into the study and will be demonstrated using two sets of images, one printed on opaque paper and the other on a transparency. Participants and their caregivers (where relevant) will be asked to immediately report any experience of diplopia to their local study team. Study clinicians will also ask participants about diplopia at each follow-up visit and repeat the diplopia demonstration. If the participant reports diplopia at any time, the condition will be assessed by study clinicians using a diplopia questionnaire [60, 61] and standard clinical techniques will be used to assess the type of diplopia. If diplopia is confirmed, the study treatment will cease and the patient will be offered further treatment if required.

#### Sample size

Thirty-six participants (18 per arm) in each of the three age groups (7–12 years, 13–17 years and 18 years and over) will provide 90 % power at *p* = 0.05 to detect a group difference of 0.20 LogMAR [62, 63] in amblyopic eye distance visual acuity change at 6 weeks post randomisation between the two treatment arms, assuming a standard deviation of 0.17 LogMAR [32] and a 10 % loss-to-follow-up at 6 weeks. Each age group has been independently powered. Overall the study will aim to recruit a total of 108 participants (54 per arm) with 36 participants (18 per arm) in each of the three age groups (7–12 years, 13–17 years and 18 years and over).

#### Analyses

During the trial, data will be entered into a secure database with range checks for each data field. Anonymised paper copies of forms will be stored in secure locked filing cabinets. The final dataset will be made available to study team members upon approval from the Steering Committee.

The effect of the intervention will be evaluated between the active and placebo groups for the whole cohort and for each of three age groups; 7–12 years, 13–17 years and 18 years and over. This design will allow for a direct comparison to the current ‘gold standard’ clinical trial of monocular amblyopia treatment in older children (7–12 and 13–17 age groups) [22].

Statistical analyses will be performed using SAS version 9.4 (SAS Institute Inc. Cary, NC, USA) and will follow a prespecified statistical analysis plan. No interim analyses are planned. All statistical tests will be two-sided at the 5 % significance level. Treatment evaluations for the primary outcome will be carried out on an intention-to-treat (ITT) basis, where the ‘last value carried forward’ method will be used to replace missing data. Sensitivity analyses will be conducted to test the robustness of the primary outcome results. These will include per protocol analysis (excluding participants who have major protocol violations such as poor compliance, loss to follow-up and missing data), complete case analysis, and ITT analyses using multiple imputation to replace missing values in the primary outcome.

Baseline demographics, visual acuity, stereopsis, suppression, and quality of life will be summarised using descriptive statistics by treatment arms. The primary outcome and quality of life data will be analysed using linear regression. Repeated measures mixed models will be applied to other continuous outcomes measured repeatedly over time and the interaction effect between treatment and visit will be tested. For all continuous outcomes the baseline outcome value and age groups (stratification factor) will be adjusted for in the regression models. The model-adjusted treatment difference will be reported, with associated 95 % confidence interval and *p* value. Generalised linear regression models will be applied to categorical outcomes as appropriate. Results will be published and presented to the relevant professional groups.

#### Monitoring

An independent monitor will check the existence and correct date for all signed Consent Forms. The monitor will also sample over 10 % of all randomised participants to check data within the database against source data. The Health Research Council of New Zealand Data Safety Monitoring Core Committee considered the trial to be low risk and, therefore, did not recommend establishing a Data Safety Monitoring Committee for the trial.

## Discussion

This is the first RCT for amblyopia in older children and adults using a binocular treatment approach. This study builds upon previous case-series studies to evaluate the efficacy of a home-based binocular videogame for improving amblyopic eye visual acuity. This study also aims to determine whether this novel treatment reduces interocular suppression and improves stereopsis.

The specific binocular videogame treatment being used in this trial is a prototype that is based on the principle of providing contrast-balanced dichoptic stimuli to patients with amblyopia in order to promote binocular function. If the treatment is effective, this general principle could be applied to a wide variety of videogame genres and platforms to enhance patient engagement and tailor the treatment to children, teenagers and adults.

### Trial status

Ongoing.

## Abbreviations

D: Dioptre
E-ETDRS: Early Treatment for Diabetic Retinopathy Study
EVA: Electronic Visual Acuity
ITT: Intention-to-treat
LogMAR: Logarithm of the minimum angle of resolution
RCT: Randomised controlled trial
VA: Visual acuity
WHOQOL – BREF: World Health Organisation Quality of Life questionnaire – Brief
